# The use of suction-assisted liposuction for lymphoedema management and rotational flap closure in a bariatric patient with lower limb open fracture

**DOI:** 10.1080/23320885.2026.2612782

**Published:** 2026-01-10

**Authors:** Rawan Jaibaji, Calver Pang, Keith Anderson, Charles Loh

**Affiliations:** ^a^ Addenbrookes Hospital, Cambridge University Hospitals, Cambridge, UK; ^b^ Department of Surgical Biotechnology, Division of Surgery and Interventional Science, Medical Sciences, University College London, London, UK

**Keywords:** Open fracture, lower limb trauma, lymphoedema, rotational flap, gustilo-anderson IIIb

## Abstract

Advanced lymphoedema and morbid obesity complicate soft-tissue reconstruction following open fractures. A 43-year-old woman with a Gustilo–Anderson IIIb ankle fracture underwent suction-assisted liposuction to reduce limb volume and improve tissue compliance, enabling tension-free rotational flap coverage. Healing was uncomplicated, with fracture union and functional recovery.

## Introduction

The process of soft tissue healing is closely linked to the restoration of lymphatic function, especially in cases of compound fractures and open wounds, where the repair mechanisms are particularly complex. Following injury, the body initiates coagulation, which is then followed by an inflammatory response involving immune cells such as lymphocytes, macrophages, and granulocytes. These cells work together to aid in tissue repair and reestablish normal physiological function. However, if the lymphatic system is compromised, it can severely impact wound healing. While lymphatic capillaries in healthy tissues possess significant regenerative abilities, their recovery in areas affected by trauma is often impaired [1]. Damage to soft tissue disrupts the microvasculature, leading to increased plasma leakage, fluid buildup in the dermis, and dysfunction of the lymphatic system [2].

Another consideration within lower limb reconstruction and that can co-exist with lymphoedema is lipoedema. Lipoedema is a chronic, progressive condition characterized by the abnormal accumulation of subcutaneous fat, primarily in the lower limbs. It is a clinical diagnosis based on clinical assessment and examination. The condition can lead to pain, bruising, and mobility issues, significantly impacting the quality of life of a patient. In true lipodema, there is no lymphatic dysfunction however in patients who may also have raised BMI there may be oedema present due to overwhelming the normal transport capacity of the cells in the body. Lower limb reconstruction often involves addressing soft tissue defects caused by trauma, infection, tumor excision, or chronic conditions. However, when lipoedema or lymphoedema coexists with these defects, it presents unique challenges that complicate both surgical planning and post-operative outcomes. Soft tissue reconstruction in patients with lipoedema or lymphoedema is complex due to fluid imbalance, poor tissue integrity, increased infection risk, and delayed healing [3].

This case report illustrates the challenges posed by lymphatic dysfunction in the setting of traumatic soft tissue injuries and highlights the essential role of lymphatic regeneration in achieving optimal healing outcomes [2]. It presents an innovative approach to managing a complex, but not uncommon, case involving a bariatric patient with a severe open ankle fracture accompanied by a significant soft tissue defect. The novel method used to facilitate soft tissue reconstruction in trauma is explored in detail in this case report.

## Patients/materials and methods

The patient, a 43 year old female, weighing 190 kg (class 3 obesity, BMI 68), presented with a low energy open bimalleolar ankle fracture following a fall from standing. She sustained a Gustilo-Anderson type IIIb injury with a significant soft tissue defect over the medial malleolus. She had a background of obstructive sleep apnoea, stage 4 chronic lymphoedema and lipoedema, exacerbated by this trauma. She is a smoker and self- reports excessive alcohol intake.

**Figure 1.**
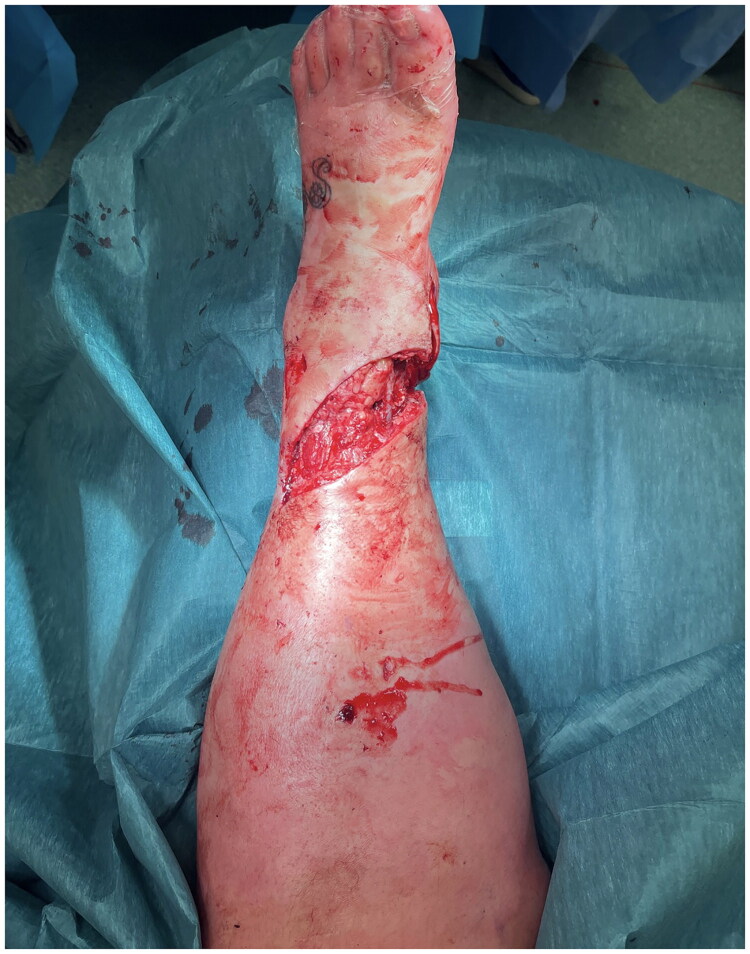
Defect following debridement of open fracture anteriorly.

**Figure 2.**
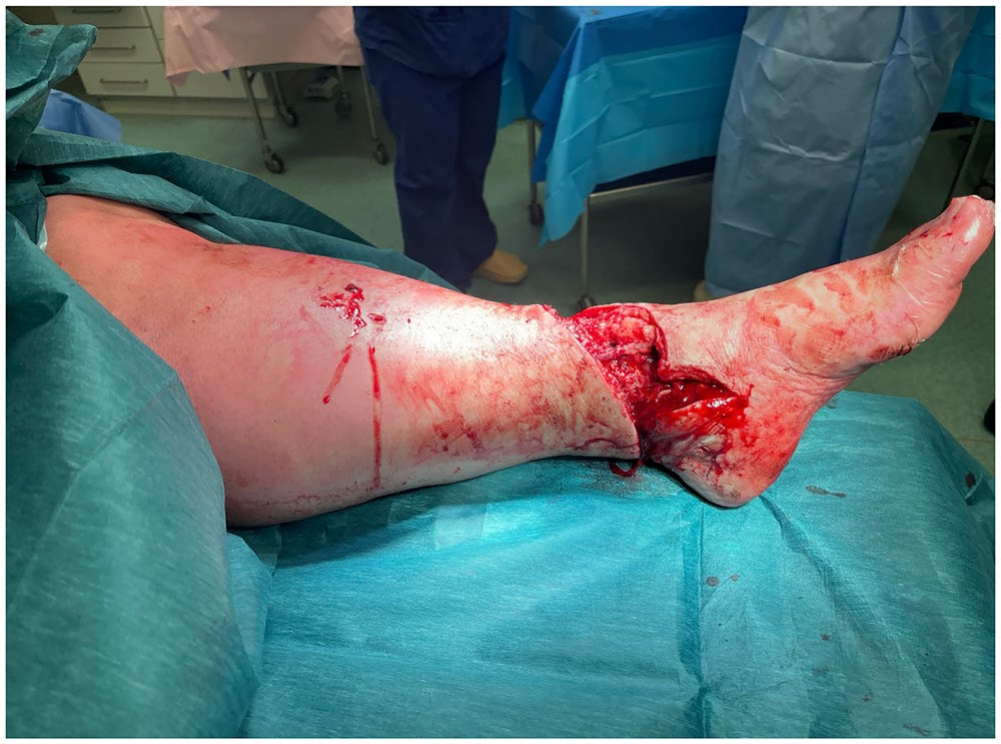
Defect following debridement of open fracture medially.

Initial debridement was performed to remove necrotic tissue and reduce the bacterial load. The fracture was then reduced, however, the primary challenge remained: the excess soft tissue and lymphedema, which precluded straightforward wound closure and increased the risk of complications such as infection, delayed healing, and non-union. The wound was too tight to close primarily and the tissues non elastic and woody, with a positive stemmer sign. Figures 1 and 2 illustrate the defect following debridement. A decision was made to perform suction-assisted liposuction (SAL) using a flying squirrel technique to the left lower leg tissue. The flying squirrel technique is a surgical method used in the treatment of advanced lymphoedema, particularly to reduce limb volume and improve skin mobility. This technique involves SAL to reduce volume and contour the limb, followed by wide skin and subcutaneous fat excision (usually if the skin can be pulled by more than 4 cm) which removes fibrotic, non-elastic tissue that restricts limb function and mobility. This resulted in a reduced volume of the surrounding soft tissue, thereby facilitating better skin mobility and reducing tension on the wound edges whilst still preserving skin perfusion. SPY fluorescent imaging technique, a fluorescence-based imaging technology used during surgery to visual blood flow and tissue perfusion in real-time, was not required in this case as the wound edges post debridement were bleeding and capillary refill was evident. Vascularity was preserved by avoiding SAL in the corridor for the posterior tibial artery perforators and focusing it on other areas for vascularity. 600mL of tumescent was administered to the left lower leg (1 mL 1:1000 adrenaline in normal saline) and a total of 500 mL of aspirate was removed (including approximately 300mL of fat).

Following debulking, a rotation flap was then raised laterally to close the defect without tension and ensuring skin perfusion is maintained. The flap provided a well-vascularized tissue to the wound site, promoting healing and reducing the risk of infection.

## Results

**Figure 3.**
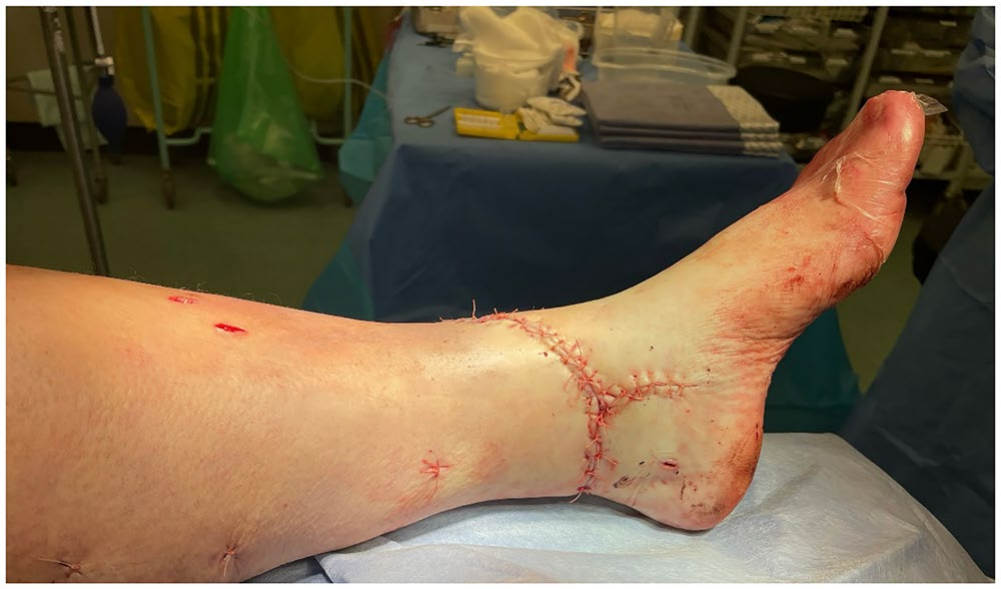
Medial view of immediate post-operative wound closure.

**Figure 4.**
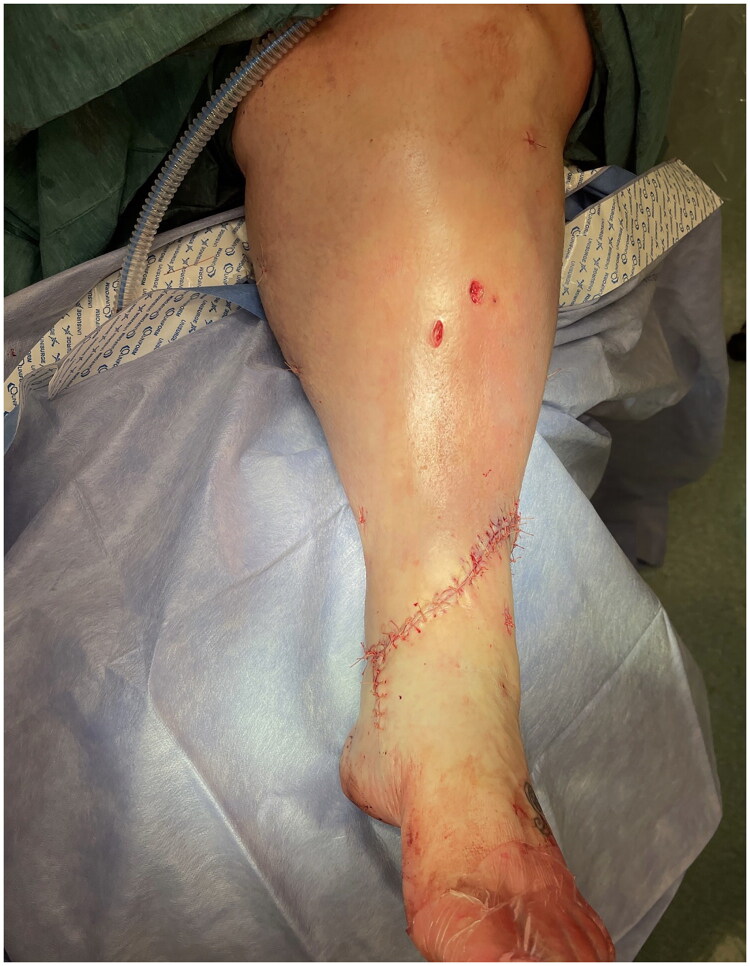
Antero-medial view of immediate post-operative wound closure.

**Figure 5.**
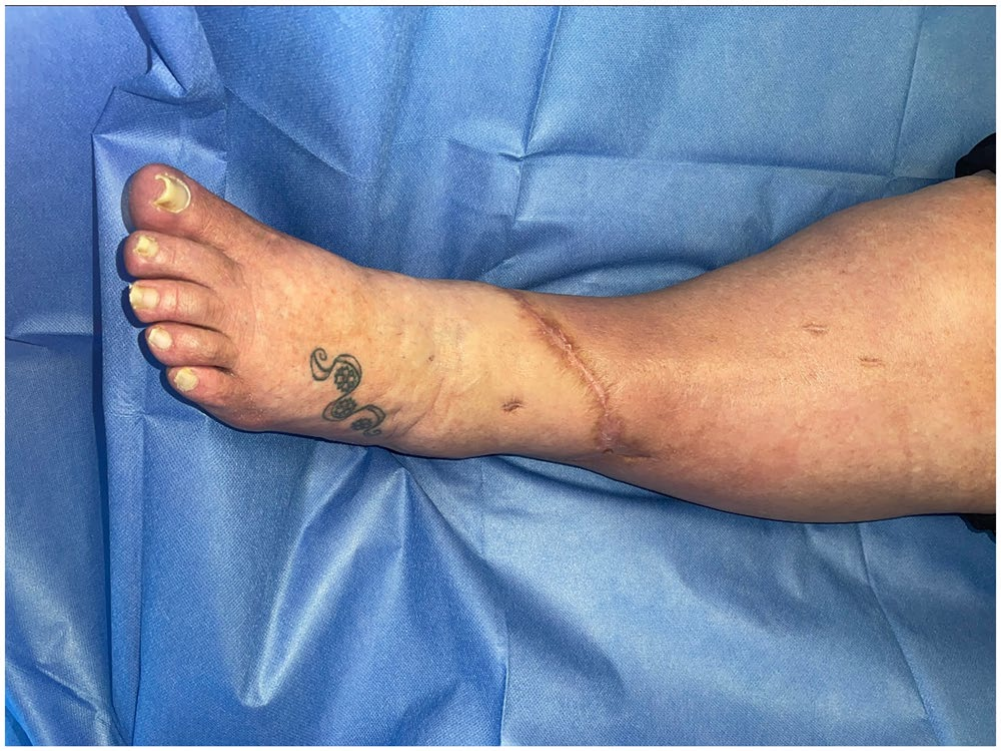
Anterior view of post operative wound at 9 months.

**Figure 6.**
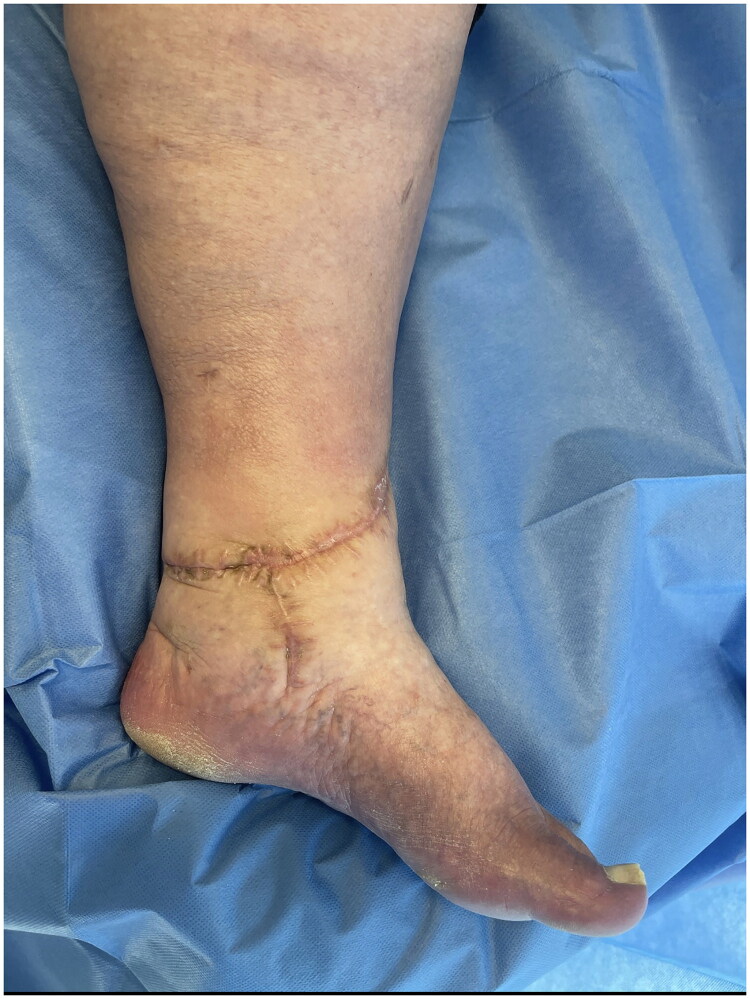
Medial view of post-operative wound at 9 months.

**Figure 7.**
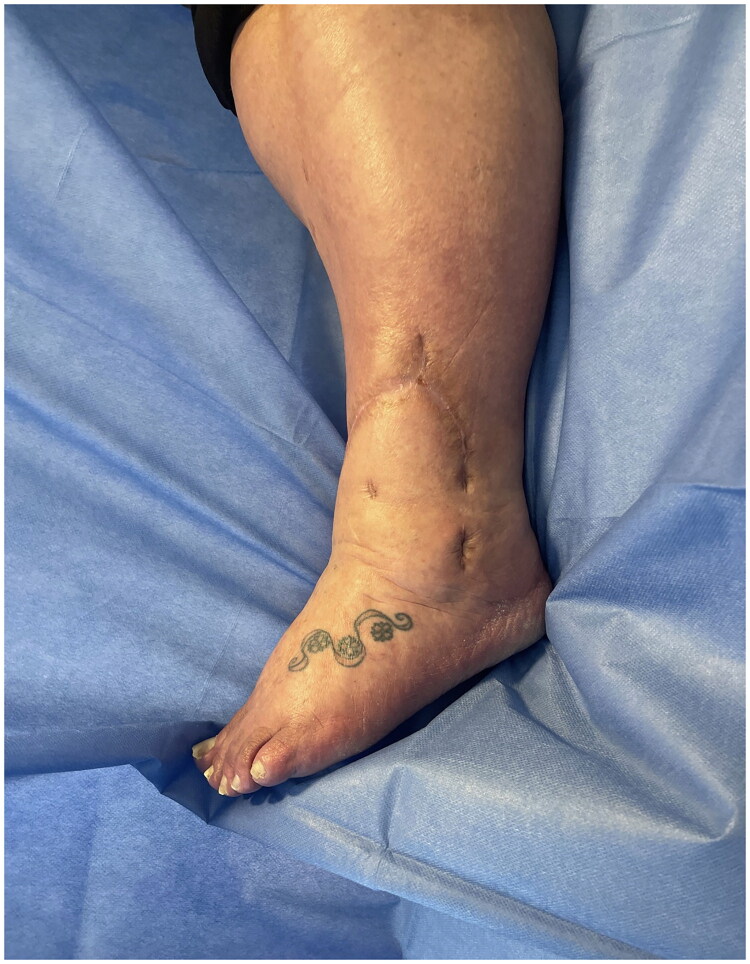
Lateral view of post-operative wound at 9 months.

Figures 3 and 4 show the immediate post-operative wound closure. Post-operatively, the patient experienced an uneventful recovery with the wound healing well with no post-operative infection or osteomyelitis and the fracture showing signs of union within the expected timeframe following 6 weeks of non-weight bearing. This patient was regularly reviewed post-operatively, with the wound pictured at 9 months in Figures 5, 6 and 7. Patient also self reports return to functional baseline (mobilising with walking stick and full weight bearing) and is pleased with the cosmetic outcome.

## Discussion

The patient’s combination of morbid obesity, chronic lymphoedema, smoking, and alcohol intake created a high-risk wound environment. Smoking-induced vasoconstriction and reduced tissue oxygenation can impair angiogenesis and increase the risk of flap necrosis. Similarly, excessive alcohol consumption is associated with immunosuppression, poor nutritional status, and delayed collagen synthesis, all of which compromise tissue repair and heighten infection risk. These factors were carefully considered in the surgical planning. Given the patient’s smoking and alcohol use, both known to impair vascular perfusion and immune function, particular attention was paid to ensuring flap viability and minimizing infection risk through volume reduction and tension-free closure [4,5].

Had this flap reconstruction failed, alternative treatment options would have been carefully considered to prevent further morbidity. Negative pressure wound therapy (NPWT) can serve as a valuable interim or definitive treatment by promoting granulation tissue formation, reducing local edema and managing exudate, which are factors especially beneficial in lymphoedematous limbs. Split-thickness skin grafting may be feasible if a healthy, well-vascularized wound bed develops, providing a less invasive alternative to further flap surgery. Allowing the wound to heal by secondary intention, often supported by NPWT would have also been considered. Skin stretching device could also be used to facilitate delayed primary closure but given the poorer quality of skin this strategy was not employed due to the risk of ischemia and wound edge necrosis. Furthermore, timely surgery given the exposed soft tissue was important and short term compression therapy was unlikely to achieve sufficient volume reduction or tissue pliability for primary closure. Free tissue transfers or pedicled muscle flaps carry higher risks in the setting of chronic lymphoedema in this patient.

There have been reports of simultaneous liposuction and skin excision in lymphoedema patients being successful in terms of reduction in complications, improvement of aesthetic outcomes, and increased patient satisfaction and the benefits of SAL in managing lower limb lymphoedema have been reported [6,7]. It has become a valuable treatment option to reduce excess volume caused by adipose and fibrotic tissue accumulation, which develops secondary to long-standing lymphatic insufficiency. SAL, a technique that uses negative pressure to remove subcutaneous fat, has been shown to significantly improve limb contour, function, and quality of life in lymphoedema patients, particularly when combined with lifelong compression therapy [8,9]. However, its impact on lymphatic structures requires careful consideration. At the macroscopic level, SAL may disrupt superficial lymphatic collectors, particularly if performed without regard to lymphatic anatomy, potentially exacerbating lymphatic drainage impairment. At the microscopic level, SAL can alter the architecture of peri lymphatic tissues and capillary integrity, although studies suggest that when performed meticulously, lymphatic function is not significantly worsened in chronic lymphoedema patients, likely because the affected lymphatics are already severely compromised, and this was a key consideration when managing this patient [10]. In fact, histological studies show that the lymphatic capillaries are often replaced by fibroadipose tissue in late-stage lymphoedema, making SAL an appropriate intervention for volume reduction [11].

This case highlights a novel method that has not been reported in the literature- utilizing SAL to address the lymphoedematous soft tissue before performing a rotational flap for wound closure in the context of open fracture lower limb trauma. This approach not only facilitated effective wound closure but also provided a new pathway for treating similar cases in bariatric patients who have open fractures where the presence of excessive adipose tissue and lymphoedema complicates traditional surgical methods. The wound defect posed a significant challenge in both wound management and overall treatment due to the presence of lymphoedema in the limb. The use of SAL not only facilitated wound closure but also contributed to a reduction in limb volume, improving the patient’s overall limb function and mobility.

The combination of liposuction with a rotational flap in this context is innovative, as it allowed for effective management of the soft tissue component of the injury while providing adequate coverage and stability to the fracture site. This successful outcome suggests that SAL, combined with a rotational flap, may be a valuable addition to the surgical repertoire for treating similar cases in the future.

## Conclusion

Given the promising results of SAL in lymphoedema management and the management of this trauma patient, further research is warranted to evaluate the combined approach of SAL followed by rotational flap closure for wound healing in lymphoedematous areas following lower limb trauma. This may initially be best approached through further case series to ascertain the success of this intervention on specific cases deemed appropriate by the operating surgeon.
